# Therapeutic implications for the PD-1 axis in cerebrovascular injury

**DOI:** 10.1016/j.neurot.2024.e00459

**Published:** 2024-10-05

**Authors:** James Feghali, Christopher M. Jackson

**Affiliations:** Department of Neurosurgery, The Johns Hopkins University School of Medicine, Baltimore, MD, USA

**Keywords:** Immune checkpoint, Ischemia, Programmed cell death protein-1, Stroke, Subarachnoid hemorrhage

## Abstract

Since the discovery and characterization of the PD-1/PD-L pathway, mounting evidence has emerged regarding its role in regulating neuroinflammation following cerebrovascular injury. Classically, PD-L1 on antigen-presenting cells or tissues binds PD-1 on T cell surfaces resulting in T cell inhibition. In myeloid cells, PD-1 stimulation induces polarization of microglia and macrophages into an anti-inflammatory, restorative phenotype. The therapeutic potential of PD-1 agonism in ischemic stroke, intracerebral hemorrhage, subarachnoid hemorrhage-related vasospasm, and traumatic brain injury rests on the notion of harnessing the immunomodulatory function of immune checkpoint pathways to temper the harmful effects of immune overactivation and secondary injury while promoting repair and recovery. Immune checkpoint agonism has greater specificity than the wider and non-specific anti-inflammatory effects of other agents, such as steroids. PD-1 agonism has already demonstrated success in clinical trials for rheumatoid arthritis and is being tested in other chronic inflammatory diseases. Further investigation of PD-1 agonism as a therapeutic strategy in cerebrovascular injury can help clarify the mechanisms underlying clinical benefit, develop drugs with optimal pharmacodynamic and pharmacokinetic properties, and mitigate unwanted side effects.

## Introduction

In 1992, the lab of Tasuku Honjo at Kyoto University identified a new member of the immunoglobulin gene superfamily while screening for genes involved in apoptosis; they referred to the gene and its protein as programmed cell death protein 1 (PD-1) [1]. Subsequently, the same group discovered the expression of this protein on the surface of stimulated B and T lymphocytes and described autoimmune phenotypes arising in PD-1 deficient mice, highlighting the role of PD-1 as a negative immune regulator [[2], [3], [4], [5], [6]]. Given the far-reaching implications of this discovery, and the successful application of this finding to cancer immunotherapy, Dr. Honjo was awarded the 2018 Nobel Prize in Physiology or Medicine alongside immunologist James P. Allison who characterized the function of another immune checkpoint molecule: cytotoxic T-lymphocyte antigen number 4 (CTLA-4) [7,8]. Programmed death-ligand 1 (PD-L1) was identified by Dong et al. in 1999 as a costimulatory molecule of T cells leading to downstream production of IL-10. As it was not known to be the ligand for PD-1 at the time, it was called B7-H1 (homology to B7-1 and B7-2 - costimulatory ligands expressed on antigen presenting cell surfaces) [9]. Further work in 2000-2001 clarified the interaction of B7-H1 with PD-1, as well as the presence of a second ligand, programmed death-ligand 2 (PD-L2) [10,11].

While the CNS was initially thought of as an immunologically isolated and privileged site due to the blood-brain barrier (BBB), permeability has been demonstrated secondary to both mechanical forces (elevated intracranial pressure, shear force) and cellular mechanisms (cytokine upregulation of endothelial adhesion molecules facilitating leukocyte diapedesis) [12,13]. Elucidation of lymphatic pathways in proximity to dural venous sinuses as well as the identification of brain antigens in deep cervical lymph nodes further highlighted the cross-talk that exists between the CNS and peripheral immune compartments underlying neuroinflammation [14,15].

The PD-1 pathway has recently been identified as an important mediator of the inflammatory response after ischemic or traumatic injury. An increased incidence of cardiovascular events, including myocardial infarctions were observed in patients taking PD-1/PD-L1 immune checkpoint inhibitors, which sparked interest in elucidating the pathway's involvement in acute coronary syndrome [16,17]. Patients with cardiac disease demonstrated higher expression of myocardial PD-L1 compared to controls [18], and the administration of PD-L1 was shown to attenuate myocardial injury in a murine model of myocardial infarction [19].

In parallel, a growing body of work has identified a role for the PD-1/PD-L1 pathway in driving neuroinflammation [13,20]. Intrathecal soluble PD-L1 administration improved motor function in a murine spinal cord injury model [21],and similar beneficial outcomes were reported in cerebrovascular injury models with IV injection. Pre-clinical evidence for positive anti-inflammatory effects of sPD-L1 via PD-1 agonism now exists for ischemic stroke [22,23], intracerebral hemorrhage (ICH) [24], subarachnoid hemorrhage (SAH)-related vasospasm [25], and traumatic brain injury [26] (TBI).

In this review, we first provide background key information on the PD-1/PD-L pathway and then focus on the role of PD-1/PD-L signaling in neuroinflammation following cerebrovascular injury and potential therapeutic implications.

## Canonical PD-1/PD-L Pathway in T lymphocytes and Antigen-Presenting Cells

**Fig. 1:**
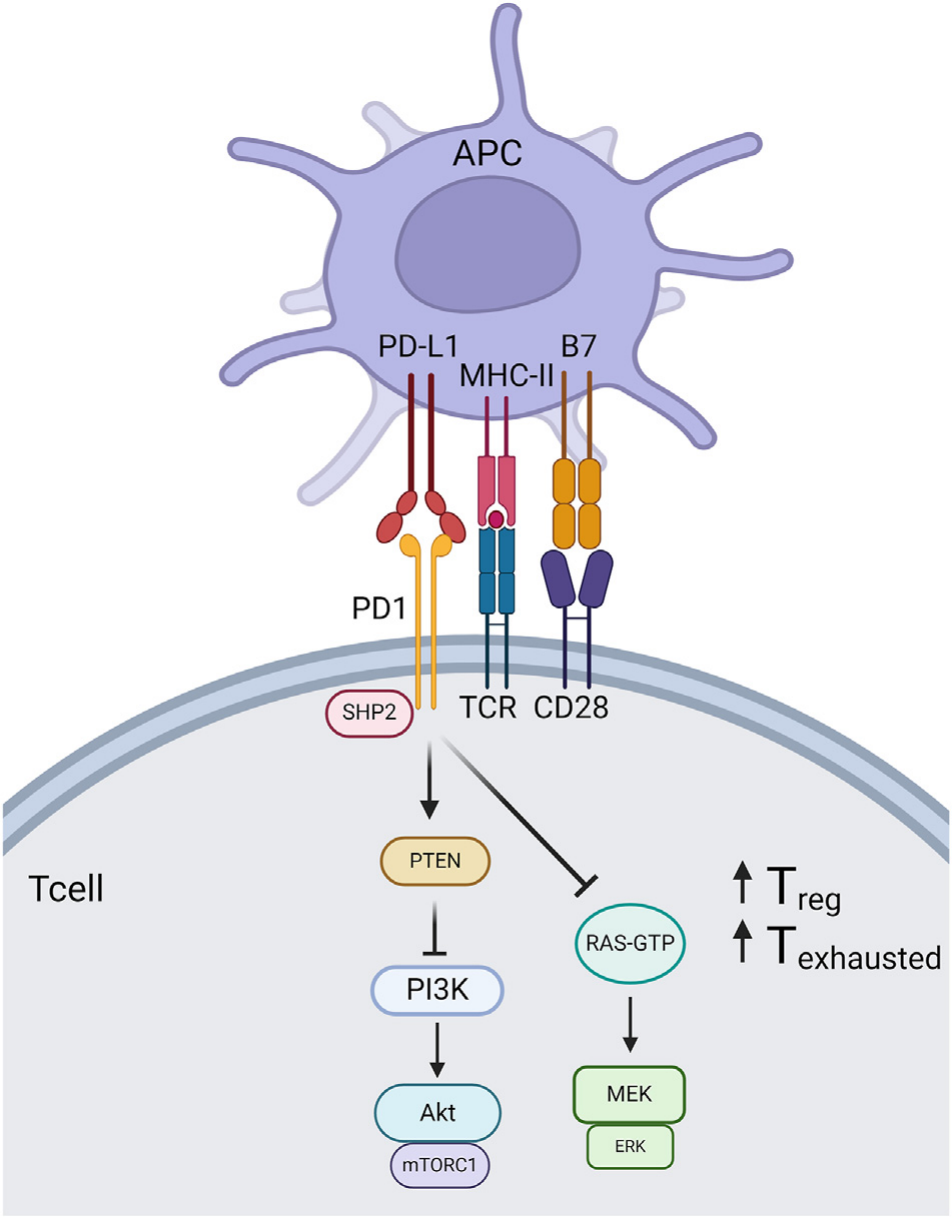
The canonical PD-1/PD-L1 pathway (created with BioRender.com).

Many early studies of PD-1/PD-L1 interactions focused on interactions of PD-1 on T cell surfaces with PD-L1 on antigen presenting cells and cancer cells [27]. Specifically, PD-1 binding PD-L1 inhibits multiple signaling pathways in the T cell, including the PI3K/Akt and Ras/MEK/Erk pathways which lie downstream to and are activated by T cell receptor (TCR) – Major histocompatibility complex II (MHC-II)/antigen interactions. This ultimately downregulates amino acid metabolism and glycolysis while promoting fatty acid oxidation, which in turn enhances T cell differentiation into regulatory or exhausted cells rather than effector or memory cells (Fig. 1) [6]. PD-1 inhibitory function is mediated by the phosphatase protein SHP2, which acts on cascades downstream of both the TCR and CD28 co-stimulator [28]. Although the effects of PD-L1 on PD-1 binding have been well defined in T cells, the responses to PD-L2 are less clear as both inhibitory and stimulatory effects have been identified [10,29].

## Soluble Forms of PD-L1

In addition to the membrane-bound form of PD-L1, multiple endogenous soluble forms of PD-L1 (sPD-L1) are generated by splicing of the corresponding membrane-bound gene or by enzymatic cleavage of membrane-bound PD-L1 [30,31]. The biological significance of endogenous sPD-L1 is a topic of active investigation. It has been shown that sPD-L1 can act as a PD-1 blocker/decoy by binding PD-1 without triggering signal transduction and simultaneously shielding it from membrane-bound PD-L1. Shi et al. were able to demonstrate that effect in their *in vitro* study of T cells in the context of atherosclerotic disease and diabetes [32]. Conversely, it may function similarly to membrane-bound PD-L1 by binding PD-1 and stimulating downstream signaling [33]. This has been borne out in oncological studies such as those of renal cell carcinoma [34] and breast cancer [35], whereby sPD-L1 inhibited T cell function by binding PD-1. A dimeric form of sPD-L1 described by Mahoney et al. was shown to inhibit T cell function more effectively than monomeric sPD-L1 [36]. Additionally, Xu et al. found that sPD-L1 interactions with PD-1 on monocyte-derived macrophages triggered apoptosis in the context of acute-respiratory distress syndrome [37]. Interestingly, Liang et al. showed that higher-affinity sPD-L1 generated by directed molecular evolution has a much slower off rate, causing lower on/off receptor interaction cycles and hence effectively acting as a decoy blocker of PD-1 [38]. This indicates that PD-1 binding affinity may influence the ultimate biological effect of sPD-L1.

## PD-1 in Myeloid Cells

More recently, the expression of PD-1 and its role in myeloid cells, especially under pathological conditions, is increasingly being recognized and studied. Microglia and macrophages can run many immunologic programs that have generally been dichotomized as M1-like (pro-inflammatory) and M2-like (anti-inflammatory). In injured tissues, these phenotypes are in a state of dynamic equilibrium; whereby, interferon-γ and lipopolysaccharide drive differentiation into the “classically activated” M1 phenotype via STAT1 while interleukin-4 and interleukin-13 promote the “alternatively activated” M2 phenotype via STAT6 [[39], [40], [41]]. PD-1 signaling increases STAT6 phosphorylation and decreases STAT1 phosphorylation, inducing M2 polarization of microglia and macrophages [42,43]. Complexity arises when one appreciates that the same cell can switch between M1 and M2 phenotypes (and likely many intermediates on this spectrum) in response to changes in the local milieu [44,45]. Of note, sPD-L1 has been shown to exert similar effects, with important physiological roles such as facilitating immune tolerance to a fetus in pregnancy as Zhang et al. found that under the influence of interferon-β, trophoblast cells secrete sPD-L1 which mediates M2 macrophage polarization via the binding of PD-1 [46,47].

Macrophage precursors (monocytes) similarly exist in multiple, dynamic states: classical (CD14^++^, CD16^−^) generally pro-inflammatory monocytes engaging in phagocytosis, intermediate (CD14^++^, CD16^+^) monocytes involved in antigen presentation and T cell activation, and non-classical (CD14^+^, CD16^++^) generally anti-inflammatory monocytes [48,49]. Corresponding monocyte subsets in mice have also been described and classified by the surface receptor Ly6C, chemokine receptor CCR2, and CD43 as follows: classical Ly6C^hi^CCR2^+^CD43^low^, intermediate Ly-C6^high^CD43^hi^, and non-classical Ly-C6^low^CCR2^−^CD43^hi^ monocytes [50,51]. Based on murine flow cytometric data, PD-L1 appears to be densely expressed characteristically by non-classical monocytes and aids in their immunomodulatory function [52].

The biology of PD-1/PD-L1 in modulating innate immune responses to acute injury is a growing topic of interest and appears to have wide-ranging implications, particularly in cerebrovascular injury, including ischemic stroke, ICH, SAH-related vasospasm, and TBI. As PD-1 agonists are currently being developed and tested in clinical trials, there is excitement regarding their potential applications to acute ischemic injury in addition to autoimmunity.

## Ischemic Stroke

Ischemic stroke or the sudden interruption of blood flow to the brain, is one of the leading causes of morbidity and mortality [53,54]. Cell death in the acute phase causes the release of damage-associated molecular patterns (DAMPs) which activate resident microglia via toll-like receptors (TLR) and trigger an inflammatory response both locally and systemically [55]. An influx of antigen presenting cells such as monocyte-derived macrophages and dendritic cells characteristically precedes neutrophil infiltration in ischemic stroke and occurs along chemokine gradients generated by damaged neurons and activated local immune cells [56]. Monocyte migration from the bone marrow to the blood followed by homing to infarcted tissue is facilitated by the CCL2/CCR2 axis [[57], [58], [59]]. Recently, trajectory analysis has indicated that blood-borne myeloid cells found in the brain after an ischemic insult maintain a distinct transcriptome compared to their counterparts in circulation, highlighting that the cellular states of these cells are determined by the local cytokine environment rather than peripheral priming [60]. An adaptive immune response follows around day 3-4, represented by CD4^+^ and CD8^+^ T cells and the more neuroprotective regulatory T cells (Tregs) [61,62]. Vasogenic edema builds up as the BBB becomes more permeable secondary to immune cell infiltration and resulting endothelial dysfunction [63,64]. Given the fixed capacity of the cranial vault, this trans-vascular fluid shift causes increased intracranial pressure and brain shift, contributing to functional impairment and mortality [65].

The role of the PD-1/PD-L pathway in ischemic stroke is complex and still being actively investigated. In 2011, Ren et al. reported increased expression of PD-1 on activated resident microglia and infiltrating macrophages in a murine middle cerebral artery occlusion (MCAO) model. PD-1 knockout mice were found to have significantly larger stroke volumes based on histology in conjunction with increased density of microglia, infiltrating T cells, neutrophils, and macrophages, as well as elevated inflammatory cytokine production by T cells (tumor necrosis factor-α and interferon- γ) [23]. Further work in 2013 showed that the transfer of B-cells secreting IL-10, an anti-inflammatory cytokine, into B-cell-deficient mice subjected to MCAO led to increased expression of PD-1 on CD4^+^ T-cells and CD11b+ monocytes in the spleen [66].

In the same year, the group's study of PD-L1- and PD-L2-deficient mice unexpectedly showed smaller infarct volumes and lower numbers of pro-inflammatory microglia, infiltrating monocytes, and CD4^+^T-cells in the ischemic hemispheres of PD-L knockout mice compared to wild-type. There were also higher levels of IL-10-producing Treg cells in the ischemic brains of PD-L1-deficient mice in conjunction with lower splenic levels of these cells [67]. A follow-up study showed that PD-L1 and PD-L2 have differential effects on T cells and regulatory B cells. Namely, PD-L2 regulates Bregs and CD4^+^ T cells whereas PD-L1 regulates CD4^+^ and CD8^+^ T cells [68]. Yet, the role of PD-L1 after stroke remains unclear as another group showed that PD-L1 mediates Treg suppression of neutrophil-derived matrix metalloproteinase-9 (MMP-9), an enzyme contributing to BBB breakdown [69]. This supported a protective role of PD-L1 in stroke mediated by Tregs, with the authors alluding to cell-specific PD-L1 effects in ischemia to potentially explain the different effects of PD-L1. It is important to note that all the aforementioned studies had utilized global gene knockout animals, which precluded the ability to discern cell-specific effects of PD-1 and PD-Ls. Another study testing the effects of intravenous injection of PD-L1 blocking monoclonal antibodies in an MCAO model showed a decrease in infarct volumes and improved neurologic outcomes. Mechanistically they found increased levels of Tregs and IL-10 in the post-stroke brains along with reduced infiltration of pro-inflammatory cells in the infarcted brain tissue [70].

More recently, our group explored whether systemic administration of sPD-L1 impacted edema and neuroinflammation in ischemic stroke. We found that PD-1 is upregulated on circulating monocytes after stroke in humans and that higher PD-1 levels generally correlated with greater edema-to-infarct ratios. In an MCAO murine model, intraperitoneal administration of sPD-L1 improved survival and decreased brain edema without impacting infarct volume. Furthermore, treatment with sPD-L1 improved certain gait parameters and long-term sensorimotor deficits. In our mechanistic experiments, treatment led to lower PD-1 positivity in brain-infiltrating monocytes and negative enrichment of inflammatory genes with markers more consistent with a non-classical phenotype in circulating monocytes. Overall, these data support the potential therapeutic potential for using sPD-L1 to reprogram circulating monocytes to an adaptive phenotype before they reach the brain, thereby mitigating secondary injury and skewing post-stroke inflammation to a restorative rather than pro-inflammatory response [22].

## Intracerebral Hemorrhage

ICH constitutes 10–15 ​% of all strokes and is associated with increased morbidity and mortality (approximately 40 ​% mortality at one month) [71,72]. Hematoma expansion and mass effect lead to primary brain injury. Subsequently, blood products such as fibrinogen cause microglial activation via TLR4 and circulating leukocyte infiltration, with improperly programmed leukocytes leading to secondary inflammatory injury [73,74]. M1 microglia produce pro-inflammatory cytokines including interleukin-1 β and tumor necrosis factor α in addition to reactive oxygen species which damage the BBB. In contrast, M2 microglia aid in the clearance of necrotic debris and blood products while triggering angiogenesis, extracellular matrix formation, and decreased edema [75]. The PD-1/PD-L pathway appears to play an important role in the ICH inflammatory cascade.

In 2016, Yuan et al. utilized PD-1 knockout mice and a murine ICH model dependent on intracranial injection of collagenase to demonstrate that bleeding increased the expression of PD-1 in perihematomal brain tissue macrophages with knockout mice exhibiting greater cerebral edema and worse neurological deficit scores than wild-type mice [24]. The following year, Wu et al. used PD-1 and PD-L1 plasmid DNA transfection in rats subjected to autologous intracerebral blood injection and showed that PD-1/PD-L1 overexpression reduced cell death and inflammation by polarizing microglia to an anti-inflammatory phenotype [76]. Similarly, Han et al. tested the effects of intraperitoneal injection of PD-L1 in a murine ICH model and found that treatment reduced bleeding and edema and decreased neurological deficits. This corresponded with an increase in Tregs and anti-inflammatory Th2 cells as well as a decrease in pro-inflammatory Th1 and Th17 ​cells. PD-L1 was shown to inhibit the mTOR pathway and administering PD-L1 blocking antibodies led to increased apoptosis and neuronal loss [77]. In 2023, Liu et al. demonstrated that electro-nape-acupuncture in a rat ICH model produced improved neurological function, decreased inflammatory cell infiltration, and increased PD-1 expression along with M2 microglial polarization [78].

## Vasospasm following Aneurysmal Subarachnoid Hemorrhage

Cerebral vasospasm occurs between 3 and 14 days following an aneurysmal subarachnoid hemorrhage (aSAH) and is the major contributor to delayed morbidity and mortality. As many as 70 ​% of patients develop angiographic vasospasm within the initial two weeks following rupture of an intracranial aneurysm. Furthermore, 20–40 ​% of individuals experience symptomatic vasospasm, resulting in clinical deterioration attributed to delayed cerebral ischemia (DCI) [79,80]. Mounting evidence implicates inflammation in the pathophysiology of vasospasm [81]. More than 50 ​% of patients present with systemic inflammatory response syndrome which correlates with the occurrence of vasospasm, hydrocephalus, and disability [82]. Acutely, cell death and blood product breakdown releases DAMPs that initiate the inflammatory cascade through TLR binding. DAMPs that have been implicated in aSAH include protein S100, extracorpuscular hemoglobin with its derivatives, and high mobility group box-1 [83]. Upregulation of adhesion molecules on endothelial cells including intercellular adhesion molecule-1 (ICAM-1) and E-selectin enable influx of circulating monocytes, macrophages, and neutrophils into the subarachnoid space [84]. Haptoglobin (Hp) acts to scavenge toxic extracorpuscular hemoglobin, and certain human polymorphisms (Hp 2-2 genotype) of the protein are less effective at binding hemoglobin. Work by Chaichana et al. showed that genetically modified Hp 2-2 mice with aSAH in fact demonstrated higher frequencies of vasospasm compared to Hp 1-1 mice [85]. Leukocyte activation, apoptosis, and degranulation produces endothelins, reactive oxygen species, free radicals, and cytokines that promote further inflammation and vasospasm [81,86]. Monocytes likely play a critical role since corresponding inflammatory indices such as the monocyte-neutrophil-to-lymphocyte ratio and lymphocyte-to-monocyte ratio are more predictive of functional outcomes following aSAH in comparison to neutrophil-based indices [87]. Several therapeutic strategies have been tested in clinical trials such as endothelin receptor antagonists, statins, magnesium, and iron chelators; however, no significant benefit has yet been demonstrated [88,89]. Additionally, anti-inflammatory drugs including steroids, NSAIDs, and cyclosporine A have limited benefit due to off-target effects [86].

Considering the important contribution of inflammation to the pathophysiology of vasospasm and the beneficial effects of PD-L1 in ICH, we investigated the role of the PD-1/PD-L pathway in SAH-related vasospasm. Intraperitoneal administration of PD-L1 in a murine ICA perforation SAH model decreased cerebral arterial wall thickness and increased lumen diameters with the effect abrogated by pre-treatment with PD-1 blocking antibodies. Upregulation of PD-1 expression after induction of SAH was identified specifically on CD45^+^, CD11b+ myeloid cells rather than other immune cells. Analysis of myeloid cells in the brain, circulation, and periphery at different time points indicated that SAH likely causes release of PD-1+ monocytes from the bone marrow which then traffic toward the brain. Moreover, PD-L1 treated animals had lower frequencies of inflammatory, Ly6c+/CCR2+ monocytes in the brain. Pre-treatment with propranolol decreased the level of PD-1+ myeloid cells in the brain, implicating a potential role for catecholamine-mediated signaling in the release and trafficking of PD-1+ monocytes to the brain following SAH. Measurement of cerebral blood velocities via transcranial doppler along with PD-1+ monocyte frequency in the peripheral blood of patients with aSAH demonstrated that increases in PD-1+ monocytes were followed by increases in vasospasm 24 ​h later [25]. These data pointed to PD-1+ monocytes as key mediators of cerebral vasospasm, encouraging further investigation into the use of PD-1 agonists in mitigating this frequent and debilitating aSAH-related complication.

## Traumatic Brain Injury

Activation of the immune system after TBI can also have detrimental and maladaptive effects. In the acute inflammatory phase, the immune cell infiltrate is dominated by a mix of classical and non-classical microglia [90,91]. This transitions into a persistent pro-inflammatory, classical M1 phenotype polarization in the chronic phase [[92], [93], [94]]. As with other models of cerebrovascular injury, M2 polarization was shown to be associated with CNS repair while increased levels of M1 phenotypic polarization were associated with greater severity of white matter injury [[94], [95], [96]]. Evidence from murine studies supports microglial depletion in the chronic inflammatory phase as a strategy for reducing neurodegeneration and neurological deficits following TBI [97]. The adaptive immune response can also have damaging effects in the chronic phase, with CD8^+^ memory T cells contributing to motor impairment. Preliminary data supports the depletion of CD8^+^ T cells as a means of facilitating neurologic recovery following TBI [98].

There has been recent interest in exploring in the role of the PD-1/PD-L pathway in the context of TBI. Chen et al. studied this pathway in a murine model of surgical brain injury and found that PD-L1 expression increased in the tissue surrounding the resection cavity. Blockade of PD-L1 additionally increased brain edema and neurological deficits while PD-L1 protein administration reduced cerebral edema. Collectively, these data indicate a putative role for PD-1 in suppressing harmful inflammation following TBI [99]. Yang et al. further showed that this checkpoint pathway may be implicated in the relative immunosuppression that occurs following TBI, making patients susceptible to infection. Specifically, they demonstrated elevated expression of PD-1 on CD4^+^ and CD8^+^ T cells in a murine model of controlled cortical injury (CCI) and that propranolol abrogated this response [100]. In the same CCI model, Gao et al. showed that PD-L1+ reactive astrocytes are enriched surrounding the location of injury, and blockade of PD-L1 led to increased injury cavity size, greater infiltration of pro-inflammatory monocytes/macrophages, and worse motor outcomes [26].

In 2023, Zhao et al. investigated the role of the PD-1/PD-L1 pathway in hippocampal neuronal function in both healthy and diseased states, including TBI. PD-1 knockout mice displayed improved long-term potentiation and memory with similar effects observed after administration of intraventricular anti-PD-1 monoclonal antibody. This benefit was exclusive in excitatory neuron-specific PD-1 knockout mice rather than microglial-specific knockout mice. Impairments in learning and memory function that occur after TBI were shown to be alleviated with PD-1 deletion or PD-1 blockade. Mechanistically, PD-L1 was demonstrated to act as a neuromodulator that suppresses hippocampal neuronal excitability (CA1 neurons) via PD-1 signaling, affecting cognitive function [101].

## Therapeutic Potential of PD-1 Agonism

Targeting the immune PD-1/PD-L axis has demonstrated success in treating advanced and refractory cancers, and PD-1 agonism is now being explored as a powerful and specific anti-inflammatory approach. PD-1 agonism has demonstrated therapeutic potential in several preclinical autoimmune disease models, including arthritis, colitis, and lupus [[102], [103], [104]]. In 2023, the first phase 2 clinical trial demonstrating a clinical benefit of PD-1 agonism was published, describing the use of peresolimab, a humanized IgG1 monoclonal antibody that stimulates PD-1, in the treatment of moderate-to-severe rheumatoid arthritis. The treatment arm received 700 ​mg of the drug intravenously once every 4 weeks and had significantly improved disease activity as judged by patient-reported symptoms and inflammatory markers at 3-month follow-up, with no difference in adverse effects compared to placebo [105]. These results have generated considerable enthusiasm for this approach. Other trials evaluating the use of rosnilimab, another PD-1 agonist antibody [106], in rheumatoid arthritis (RENOIR trial NCT06041269) and ulcerative colitis (ROSETTA trial NCT06127043) are underway or in preparation. Helou et al. developed a PD-1 agonist that was able to downregulate innate lymphoid cell effector function and ameliorate airway hyperreactivity in a humanized mouse model of asthma [107]. Furthermore, PD-1 agonism was demonstrated to dampen airway hyperreactivity in a murine model of neutrophilic asthma by reprogramming pulmonary effector T cells [108]. This opens the path to study the use of immune checkpoint agonism in the treatment of allergic diseases.

We and others have demonstrated robust pre-clinical responses to PD-1 agonism in treating ischemic stroke and SAH-related vasospasm [22,25]. Similar data exist to support further clinical studies on PD-1 agonism in ICH [104]. In this context, the main target cells appear to be myeloid cells, specifically monocytes and macrophages, whereby PD-1 agonism can tilt the balance from a pro-inflammatory toward an anti-inflammatory phenotype that promotes repair and facilitates recovery after injury. The mechanisms underlying this activity are not fully elucidated and are a topic of active investigation.

Biodesign studies are underway to develop safe and effective PD-1 agonists. Some approaches may represent an improvement over traditional agonist antibody design. Bryan et al. were able to computationally design a hyperstable miniprotein, PD-MP1, that in a trimeric form effectively inhibits T cell activation by up to 70 ​% through binding of PD-1 [109]. Curnock et al. was able to design a cell-targeted bispecific PD-1 agonist that suppresses autoreactive T cells in a manner that mimicked endogenous PD-1/PD-L1 interactions. This restricts T cell suppression to the site of action (eg. pancreas), thereby limiting unwanted off-target effects. The agonist was referred to as PD-1 agonist ImmTAAI (immune modulating monoclonal TCR against autoimmunity) and is able to bind pancreatic cells by recognizing target cell peptide-human leukocyte antigen complexes via a T cell receptor end while binding PD-1 on T cells via their recombinant PD-L1 end [110]. As more data emerge on the potential to alleviate secondary immune damage following cerebrovascular injury through PD-1 agonism and key players and pathways in the innate immune system are better characterized bespoke PD-1 agonists to this purpose may yield optimal results. The variety of PD-1 agonist agents that have been developed with intention for clinical use are summarized in Table 1.

**Table 1:** Select PD-1 agonists in development.

Name	Publication year	Class/design	Condition tested
PD-MP1	2021	Hyperstable 40-residue miniprotein	None
PD-1 ImmTAAI	2021	Recombinant PD-L1 or anti-PD-1 agonist antibodies fused to TCR recognizing a target cell peptide-HLA complex	None
Peresolimab	2023	Humanized IgG1 monoclonal antibody	Rheumatoid arthritis phase 2 trial
Rosnilimab	2024	Humanized IgG1 monoclonal antibody	Tested on healthy volunteers, plans for RA & UC phase 2 trials

RA:Rheumatoid arthritis; UC: Ulcerative colitis.

As a new class of therapeutic, PD-1 agonists require considerable safety scrutiny. This immune checkpoint pathway has been implicated in the relative immunosuppression that is seen in critically ill patients with cerebrovascular injury such as TBI and ICH [100,111]. Following the release of the peresolimab trial, there were also expressed concerns about a potential increased risk of cancer similar to what was seen with tofacitinib, a Janus kinase inhibitor used for rheumatoid arthritis. Although a phase 2 trial cannot evaluate such risk, post-marketing surveillance studies with larger samples may uncover this other unwanted side-effect of PD-1 agonism [112,113].

Although this review focused on the PD-1/PD-L pathway, other immune checkpoint pathways have been implicated in neuroinflammatory disorders, including TIM-3 and CTLA-4 [13]. Further investigations focusing on the interplay of various immune checkpoints and their relative contribution to the pathophysiology of cerebrovascular injury can help guide future combinatorial therapeutic approaches, analogous to combination immune checkpoint inhibitor regimens in cancer.

The PD-1/PD-L immune checkpoint pathway appears to play an important role in preventing maladaptive immune activation and secondary damage following cerebrovascular injury. While the adaptive immune system and lymphocytes are the major cells regulated by this pathway in the context of cancer, the innate immune response predominates in inflammatory damage after acute cerebrovascular injury. PD-1 agonism has been shown to polarize monocytes and macrophages toward an anti-inflammatory phenotype, limiting secondary brain injury and promoting recovery. PD-1 agonism is emerging as a promising therapeutic strategy to treat ischemic stroke, ICH, vasospasm, and TBI. Furthermore, available data indicate that PD-1 upregulation on innate inflammatory cells may be a conserved response to ischemia, indicating potential therapeutic activity in myocardial infarction and other ischemic pathologies. Meanwhile, additive or synergistic effects with other known immune checkpoints affords the potential of fine tuning immune responses analogous to combination immune checkpoint blockade in cancer.

## Author Contributions

J.F. wrote the manuscript. C.M.J. conceived of the topic and outline for the review, edited the manuscript, and approved the final version of the manuscript.

## Declaration of competing interest

The authors declare the following financial interests/personal relationships which may be considered as potential competing interests: Christopher M. Jackson reports financial support was provided by Grifols Inc. Christopher M. Jackson reports financial support was provided by InCephalo. Christopher M. Jackson reports financial support was provided by Biohaven Ltd. Christopher M. Jackson reports a relationship with Egret Therapeutics that includes: equity or stocks. Christopher M. Jackson has patent pending to Johns Hopkins University School of Medicine. If there are other authors, they declare that they have no known competing financial interests or personal relationships that could have appeared to influence the work reported in this paper.
